# Comparison of Unsupervised Machine Learning Approaches for Cluster Analysis to Define Subgroups of Heart Failure with Preserved Ejection Fraction with Different Outcomes

**DOI:** 10.3390/bioengineering9040175

**Published:** 2022-04-16

**Authors:** Hirmand Nouraei, Hooman Nouraei, Simon W. Rabkin

**Affiliations:** Department of Medicine, Division of Cardiology, University of British Columbia, Vancouver, BC V5Z 1M9, Canada; hnouraei@gmail.com (H.N.); h.nouraei@mail.utoronto.ca (H.N.)

**Keywords:** heart failure, preserved ejection fraction, unsupervised machine learning, cluster analysis

## Abstract

Heart failure with preserved ejection (HFpEF) is a heterogenous condition affecting nearly half of all patients with heart failure (HF). Artificial intelligence methodologies can be useful to identify patient subclassifications with important clinical implications. We sought a comparison of different machine learning (ML) techniques and clustering capabilities in defining meaningful subsets of patients with HFpEF. Three unsupervised clustering strategies, hierarchical clustering, K-prototype, and partitioning around medoids (PAM), were used to identify distinct clusters in patients with HFpEF, based on a wide range of demographic, laboratory, and clinical parameters. The study population had a median age of 77 years, with a female majority, and moderate diastolic dysfunction. Hierarchical clustering produced six groups but two were too small (two and seven cases) to be clinically meaningful. The K-prototype methods produced clusters in which several clinical and biochemical features did not show statistically significant differences and there was significant overlap between the clusters. The PAM methodology provided the best group separations and identified six mutually exclusive groups (HFpEF1-6) with statistically significant differences in patient characteristics and outcomes. Comparison of three different unsupervised ML clustering strategies, hierarchical clustering, K-prototype, and partitioning around medoids (PAM), was performed on a mixed dataset of patients with HFpEF containing clinical and numerical data. The PAM method identified six distinct subsets of patients with HFpEF with different long-term outcomes or mortality. By comparison, the two other clustering algorithms, the hierarchical clustering and K-prototype, were less optimal.

## 1. Introduction

The recognized heterogeneity in various clinical conditions has led to pleas for the utilization of data analytic techniques to more precisely define patient groups to customize or personalize treatment [1]. Cluster analysis has been championed as a methodology to aid in the identification of patient subtypes in complex data sets, which may overstretch a human’s capacity to evaluate such data. One condition that has challenged the field of cardiovascular disease is heart failure with preserved ejection (HFpEF). This condition affects nearly half of all patients with heart failure (HF) [2,3] and appears to have an extremely heterogeneous pathophysiology [4,5]. HFpEF has been resistant to conventional therapies, which have been successful in the treatment of other kinds of HF. The absence of an array of effective therapies accounts, in part, for a reported 50% of mortality over 5 years for HFpEF and an annual mortality of 29% after an acute decompensated HF admission [6,7,8]. The challenges in treating HFpEF underscore the importance of translating the heterogeneity of its pathophysiology into clinically identifiable phenotypes.

Novel methods, such as machine learning (ML) and specifically cluster analysis, have been proposed to aid in the understanding of this cardiovascular condition [9]. Unsupervised ML seeks inherent patterns in large complex datasets without prior knowledge of the outcome [10]. Cluster analyses, such as hierarchical clustering, K-prototype and partitioning around medoids (PAM), are important unsupervised ML techniques [10,11,12]. Each of these commonly used approaches has its own strengths and weaknesses, necessitating comparative studies for optimal algorithm selection. However, there is a lack of published comparative data analytics in healthcare and specifically in HF that usually apply only one clustering algorithm [13]. The objective of this study is to compare these three different unsupervised ML approaches to the analysis of a single mixed dataset of outpatients with HFpEF in order to determine which analytical methodology provides a better informatic approach to the identification of patient subsets with different outcomes.

## 2. Materials and Methods

### 2.1. Study Population

The study population has been described in detail previously [14]. Briefly, it was a retrospective study of 196 consecutive patients with HFpEF assessed at an ambulatory cardiology clinic. The study was approved by the ethics department at the University of British Columbia. The inclusion criteria were adults over the age of 18, in whom HFpEF was confirmed on a transthoracic echocardiogram (TTE). HFpEF was defined based on the 2016 European Society of Cardiology criteria that specified a left ventricular ejection fraction (LVEF) ≥50% [15].

### 2.2. Machine Learning

Cluster analysis identifies similar subjects based on their combined features. Similar to other studies applying artificial intelligence to HFpEF, we identified variables that were highly correlated, with Pearson’s correlation coefficient of >0.6 and removed them from the analysis [16,17]. We removed medications from the cluster analysis as they were strongly correlated with the medical conditions that were already in the dataset. Variables with more than 1% missing data were removed from the clustering analysis. Imputation using a Stochastic Gradient Descent [18] algorithm replaced a total of 10 missing values in the entire dataset. A total of three different approaches to cluster analysis were applied to the remaining 47 candidate variables. Normally distributed continuous variables were described as mean and standard deviation, and others were expressed as medians and interquartile ranges. A *p*-value of <0.05 was considered statistically significant. All analyses were performed with RStudio version 1.2 (RStudio Inc., Boston, MA, USA).

Hierarchical clustering utilizes a tree-like structure, dendrogram, to identify clusters closest to each other and distinct subgroups [10]. The Euclidean distance, a common metric in hierarchical clustering, was used to determine the separation between the subgroups [19,20,21]. The second method was a non-hierarchical iterative clustering algorithm, K-prototype. This algorithm integrates the K-means, for numerical variables, and K-modes, for categorical variables, algorithms to cluster data with mixed numeric and categorical values [11]. It uses an iterative process to create ‘k’ number of centroids, where each data point is assigned to its closest centroid, and groups that have a high similarity are defined [11]. The third method, partitioning around medoids (PAM), is another non-hierarchical iterative method that assigns ‘k’ random entities to be medoids, the most centrally located objects in each cluster. PAM is more robust than K-prototype [12,22]. Another difference between K-prototype and PAM is the distance metric used. PAM uses a Gower distance metric to measure the similarity between variables. The Gower distance selects a particular distance metric that suits each variable type and scales the results to fall between zero and one [12].

As part of the internal validation, the commonly used silhouette width, connectivity index, and the Dunn Index were evaluated for each clustering technique to arrive at the optimal number of clusters. A silhouette plot was used to determine the optimal number of clusters. This tool employs an aggregated measure of similarity between observations within a cluster and compares it to observations in neighboring clusters [23]. The number of clusters resulting in the largest silhouette width is typically the recommended choice. Silhouette width is an index reflecting the compactness of clusters and their separation from each other [23]. The Dunn index identifies clusters that are compact, with a small variance between members of the cluster and the connectivity index evaluates the inter-consecutiveness of the members of the cluster. T-distributed stochastic neighbor embedding (t-SNE) was used to visualize the high-dimensional data by giving each data point location on a two-dimensional plot.

## 3. Results

**Figure 1:**
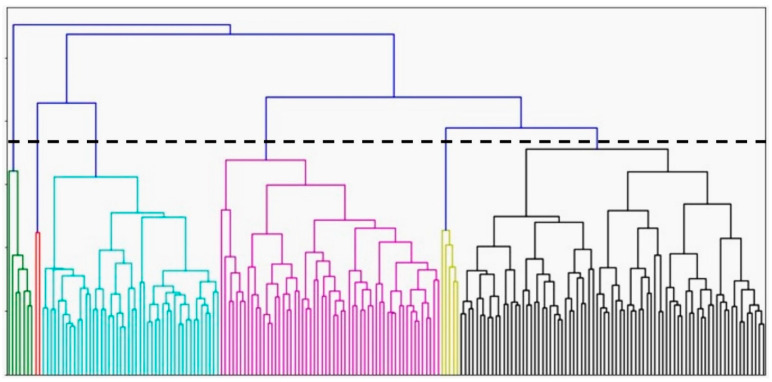
Dendrogram for hierarchical clustering (*n* = 196). Each color represents once cluster (cluster 1: green, cluster 2: red, cluster 3: blue, cluster 4: pink, cluster 5: yellow, cluster 6: black).

The hierarchical clustering method revealed the underlying architecture of the dataset and generated six different clusters. The optimal number of clusters was determined by the dendrogram and the Euclidean distance between groups (Figure 1). They also achieved better connectivity and a higher Dunn Index. Three larger clusters were found with 46 (Cluster 3), 49 (Cluster 4), and 69 (Cluster 6) members. Three smaller clusters with 23 (Cluster 1), two (Cluster 2), and seven (Cluster 5) members were also identified. There were significant differences among the clusters in features such as age, gender, diabetes, dyslipidemia, and coronary artery disease (CAD) (Table 1). Although the outcomes demonstrated statistical significance, they are not applicable clinically given the small size of two of the clusters.

**Figure 2:**
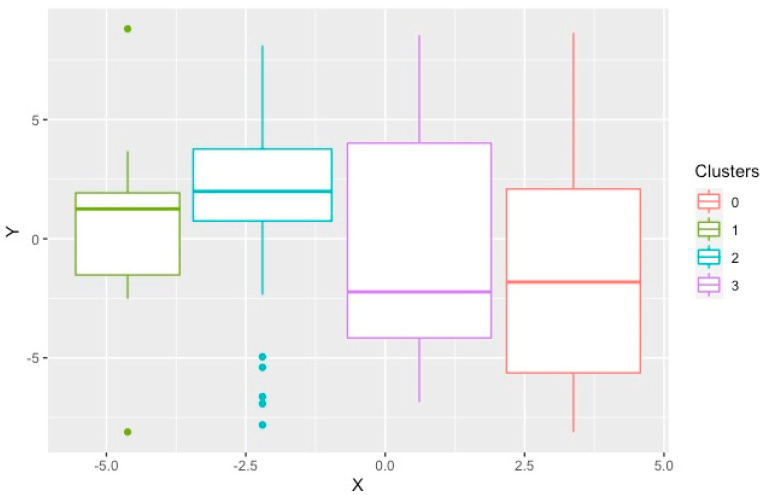
T–distributed stochastic neighborhood embedding used to show the local structures of the clusters produced by K–prototype on a two-dimensional plot. Each point represents one study subject.

In the K-prototype method, utilizing the optimal silhouette width, four different clusters were found. This was also signaled by the slightly better connectivity and Dunn Index compared to the other cluster sizes using this method. Figure 2 demonstrates the t-SNE plot for the clusters produced by K-prototype. Cluster 2 was the smallest cluster with nine members. The age and gender of subjects in these clusters were significantly different with Cluster 4 having the youngest members (Table 2). Clusters 1 and 3 consisted of predominantly females while Clusters 2 and 4 were predominantly male. Comparing comorbidities, only atrial fibrillation (AF) and chronic kidney disease (CKD), defined as estimated GFR < 60 mL/min/1.73 m^2^, were significantly different among the clusters. Systolic blood pressure (SBP) and serum low-density lipoprotein cholesterol (LDL-c) showed significant differences. Cluster 1 had the highest SBP, and Cluster 4 had the highest LDL-c. Serum BNP was significantly higher in Cluster 2 compared to the others. There were significant differences among the clusters on TTE, specifically LVEF, mitral valve E/A ratio, average E/e’ ratio and pulmonary artery pressure. The presence of elevated filling pressure was significantly higher in Cluster 2 and lowest in Cluster 4. The degree of diastolic dysfunction was significantly higher, and more likely to be moderate or severe, in Clusters 2 and 1 while most likely to be mild in Cluster 4. The Meta-Analysis Global Group in Chronic Heart Failure (MAGGIC) risk score was significantly higher in Cluster 3 compared to the other clusters. With respect to outcomes, HF exacerbation was highest in Cluster 1, whereas cardiovascular mortality, all-cause mortality, and the composite of endpoints were highest in Cluster 2.

**Figure 3:**
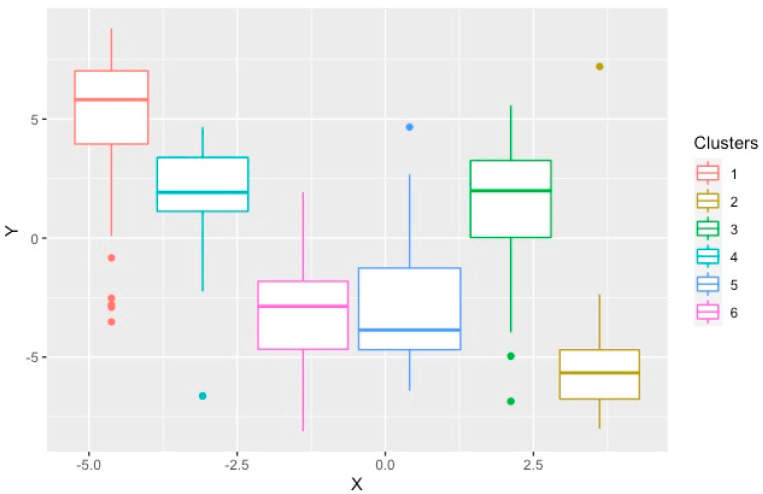
T–distributed stochastic neighborhood embedding used to show the local structures of the clusters produced by PAM on a two–dimensional plot. Each point represents one study subject.

The PAM method generated six significantly different clusters, which were well-separated as demonstrated by the optimal silhouette width, connectivity index and Dunn Index. The features of these six subgroups were compared in detail [14]. Briefly, there were three groups of women, those with a low proportion of vascular risk factors (HFpEF_1_), individuals with a high proportion of hypertension and diabetes (HFpEF_3_), and older individuals with high rates of atrial fibrillation and chronic kidney disease (HFpEF4). The other clusters were mostly men with a high proportion of coronary artery disease, dyslipidemia and diastolic dysfunction (HFpEF_2_), those with the highest BMI, obstructive sleep apnea and poorly controlled diabetes (HFpEF_5_), and individuals with high rates of AF, elevated BNP and biventricular remodeling (HFpEF_6_) [14]. Figure 3 demonstrates the t-SNE plot for the clusters produced by PAM.

## 4. Discussion

This is the first study, to our knowledge, to compare different clustering algorithms using the same dataset of outpatient subjects with HFpEF. Hierarchical clustering was the first method used to separate patients with HFpEF. Hierarchical clustering, visualized by dendrograms, produces a single nested hierarchy from which a partition can be obtained for any possible choice of the number of clusters [24]. While this feature makes hierarchical clustering popular, it is also a challenge to interpret whether the identified clusters represent an important underlying structure or are artifacts of natural sampling variation [24]. This method is limited by its experimental approach and the arbitrary determination of the number of clusters based on the resulting dendrogram and is consistent with the contention of others [16]. In our study, in addition to encountering this limitation, the cluster sizes were widely different from each other. Two of the smallest clusters had only two (Cluster 2) and seven (Cluster 5) members. Having such small clusters could indicate that there was a significant separation distance between each of these clusters and the larger ones. Comparing the clinical and echocardiographic features of these two clusters with their neighbors, however, fails to show any important features that justifies having these two groups. In fact, the more likely explanation is that Cluster 2 and Cluster 5 contain members that lie on the border of their neighboring clusters or are borderline outliers. Thus, the hierarchical algorithm, which works well when large separation distances exist between clusters [19,20,21], may be artificially forming these smaller subgroups. In fact, if a slightly larger cut-off of Euclidean distance was used, by moving the horizontal line on the dendrogram higher, these smaller clusters would have been joined with larger groups. The challenges posed by identifying the optimal number of clusters using the hierarchical method and our clinical understanding of the results conclude that this method is not optimal.

The K-prototype method produced four different clusters. We found some similarities between these clusters and the four groups of HFpEF identified using a similar algorithm, K-means, by Harada et al., in the outpatient setting [25]. In that study, similar demographics and clinical information were used with the difference that clinical outcomes were also applied to assist in forming the clusters. Categorical variables had to be converted to numerical ones due to the inherent limitations of the K-means algorithm. Another limitation of this method was that the K-means and K-prototype algorithms require an estimate of the number of clusters that naturally exist in the data and cannot identify the optimal number of clusters [25]. This limitation opens the door for random errors and biases in this clustering approach. Group 1 with younger patients and LV relaxation abnormality had the lowest mean mitral E/e′ ratio and was most similar to Cluster 4. Group 2 with older patients with renal dysfunction was similar to Cluster 2. Group 3 with AF and advanced biventricular diastolic dysfunction was similar to Cluster 1. Group 4 with older patients, renal dysfunction and had a high PASP was similar to Cluster 3. The K-prototype method discovered some underlying data structure; however, several clinical and biochemical features did not show statistically significant differences. Additionally, there was significant overlap between the clusters as demonstrated graphically (Figure 2). Thus, this approach did not satisfy the aim of identifying optimized HFpEF clusters.

Utilizing the PAM method, we discovered six distinct clusters in our database [14]. The clustering results using the PAM method not only showed significant clinical features, but also had better overall internal validity, including increased cluster compactness and separation distance. In contrast, the hierarchical and K methods produced a subgroup that consists of only 5% or less of the population thereby suggesting a less meaningful group. We used measures of internal validity as mentioned in the methods section to arrive at the optimal number of clusters within each clustering technique and did not utilize these to compare the different methods.

Few studies have conducted head-to-head comparison of different clustering algorithms on the same dataset. In a study by Bose and Radhakrishnan, three different unsupervised ML algorithms (hierarchical, K-means, and PAM) were used to identify subgroups of outpatients with HF [26]. They concluded that hierarchical clustering was the best technique using internal validation methods. There are several limitations to that study. First, from the perspective of the study of HFpEF, it did not distinguish HFpEF from HFrEF, and no laboratory or echocardiographic features were used for clustering. Instead, focus was placed on medical history, symptoms and quality of life. By performing feature selection, the number of variables used for identifying the potential clusters was reduced from nearly 300 to only seven. This was performed using a software package to find the “best fit” features for the data [26]. The drastic reduction in the number of features and use of internal validity instead of clinical outcomes for comparing the clustering techniques limit the reliability of their conclusion.

In another study, Preud’homme et al. used three unsupervised ML algorithms (hierarchical clustering or PAM, K-prototypes) to compare their efficacy [27]. These methods were first applied to a dataset of virtual populations and subsequently to the dataset from the EPHESUS randomized clinical trial, which only included patients with HFrEF [27]. For the latter analysis, the number of clusters was fixed at four for all the different algorithms allowing minimal flexibility. The authors concluded that K-prototype was dominant between the unsupervised ML methods compared. The conclusion was based on comparing the clusters from different methods using the adjusted rand index, which is a measure of similarity between clusters [27]. We believe arbitrarily setting the number of clusters to four is a major limitation in this study because it introduces significant bias and restricts the algorithms from identifying the optimal number of clusters. Outcome data were also not used to compare how the clusters differ from each other. Furthermore, that study is likely not relevant to HFpEF since all subjects had HFrEF.

Our analysis supports the contention that multiple relevant partitions can be found in a population, and cluster analysis can be considered successful if it makes sense to the practitioner specialized in the field [27]. Our study is the first, to our knowledge, to compare different clustering algorithms using the same dataset of outpatient subjects with HFpEF to identify the most suitable algorithm yielding clinically relevant results in this population.

While some might argue that heart failure is a clinical entity in which the clinician’s role is critical in the diagnosis, and there is no need for the application of machine learning to define subsets of patients, it is important to underscore the heterogeneous etiology of HFpEF which demands characterization. HFpEF has been attributed to a diverse range of abnormalities of cardiomyocyte structure and function, cardiac fibrosis, myocardial extra-cellular matrix, and vascular function [4,5]. Each of these distinct pathophysiologic entities may have different manifestations and their definition will eventually permit a tailored approach to the treatment or implementation of a precision medicine approach to the treatment of HFpEF.

Several studies have applied machine learning approaches to search for subtypes or different phenogroups in patients with HFpEF. Segar et al. examined a subset of 654 participants in the TOPCAT (Treatment of Preserved Cardiac Function Heart Failure with an Aldosterone Antagonist) study [17]. They also removed variables with a correlation coefficient >0.6, keeping the variable that was most clinically meaningful. They identified three mutually exclusive phenogroups of HFpEF participants using penalized finite mixture model-based clustering analysis on 61 mixed-data phenotypic variables [17]. Kao et al. examined data from patients with HFpEF in the I-PRESERVE (Irbesartan in Heart Failure With Preserved Systolic Function) study and considered eleven prospectively selected clinical features [28]. Data analysis used the *polkas* library in the R statistical package and Latent class analysis (LCA) definitions were derived using maximum-likelihood estimation to determine the most likely subgroup for each patient [28]. They identified six phenogroups [28]. Hedman et al. evaluated data from 320 patients with HFpEF clustering 32 echocardiographic and 11 clinical or laboratory variables from the Karolinska-Rennes cohort (KaRen study) [29]. Model-based clustering of standardized variables was performed using the *Mclust* function and the optimal model and number of clusters was determined by the maximum BIC with three multinomial classification methods in the R statistical package [29]. They identified six composite phenogroups [29].

## 5. Limitations

There are several limitations of the study that have been previously discussed in detail [14] and some warrant further comment. ML algorithms handling small sample sizes with a large number of variables can run into dimensionality and overfitting [13]. Thus, we reduced the number of highly correlating variables to minimize the effects of dimensionality. Second, the number of patients studied is small. However, the data collection allowed for a detailed description of each person. Third, we used only three different ML methodologies so that we could readily compare them. Other approaches exist and warrant comparison but increasing the number of methods would be more challenging to compare. Lastly, defining the best ML method for defining subsets of patient phenotypes can be challenging. However, we contend that a method that defines a subgroup consisting of only 5% or less of the population does not identify a meaningful group. We selected the PAM method not only because it avoided small and less meaningful subgroups, but because it also identified subgroups with significant clinical features and had better overall internal validity, including increased cluster compactness and separation distance. The computational time of the different methods were not recorded and could not be used as an index for comparison.

## 6. Conclusions

This is the first study, to our knowledge, to conduct cluster analysis on outpatients with chronic HFpEF, an entity with a high mortality and resistant to most current therapies, utilizing and comparing three different unsupervised ML approaches. The PAM method was found to perform in a robust fashion for this mixed dataset identifying six different subtypes of HFpEF_1-6_ that were found to be discrete when assessed on independent clinical outcomes. In contrast, hierarchical clustering and the K-prototype methods did not yield clusters that were clinically as distinct. This study demonstrates the need for careful evaluation of data analytic techniques in machine learning when applied to clinical medicine.

## Figures and Tables

**Table 1:** Summary of clusters using the hierarchical method (*n* = 196).

Clusters		1	2	3	4	5	6	*p*-Value
Number of subjects		23	2	46	49	7	69	
Age (years)		80 (72, 84)	76 (75, 77)	78 (70.5, 83)	68 (59, 72)	83 (68, 89)	83 (71.5, 88)	<0.001
Male (%)		13	0	57	73	86	23	<0.001
Atrial fibrillation (%)		17	0	24	14	43	35	0.100
Hypertension (%)		65	100	83	73	86	68	0.410
Dyslipidemia (%)		48	50	72	63	29	32	<0.001
Diabetes (%)		9	0	43	27	14	16	0.006
Coronary artery disease (%)		17	50	57	43	29	26	0.007
Chronic kidney disease (%)		4	0	22	12	29	26	0.153
Stroke or transient ischemic attack (%)		0	50	7	4	14	14	0.046
Obstructive sleep apnea (%)		0	0	13	8	14	6	0.447
Lung disease (%)		17	0	13	4	0	6	0.260
Body mass index (kg/m^2^)		23.1 (21, 26.6)	30.6 (22, 39.1)	25.9 (24.1, 28)	28.1 (24, 32.7)	38.5 (38, 38.5)	24.6 (22.5, 28)	0.004
Systolic blood pressure (mmHg)		139 (129, 150)	142.5 (140, 145)	136.5 (123, 153)	130 (120, 130)	123 (101, 136)	134 (120, 144)	0.055
Low-density lipoprotein (mmol/L)		2.4 (2, 3.4)	2.7 (2, 3.3)	1.6 (1.0, 2.2)	2.0 (1.5, 2.7)	2.2 (1.2, 2.5)	2.1 (1.7, 2.8)	<0.001
Serum creatinine (mmol/L)		69 (62, 96)	81.5 (67, 96)	94.5 (79, 119)	90 (73,102)	94 (91,155)	87 (71, 133)	0.012
HbA1c (%)		5.8 (5.6, 6.1)	9.1 (5.6, 12.6)	6 (5.8, 6.7)	6 (5.6, 6.7)	5.8 (5.6, 6.4)	5.8 (5.5, 6.2)	0.111
Left ventricular ejection fraction (%)		60 (56, 61)	60 (55, 65)	55 (51.8, 60)	60 (53, 60)	57 (55, 60)	60 (55, 65)	<0.001
Right ventricle diameter (mm)		30 (28, 33)	37 (37, 37)	35 (31.8, 38)	34 (32, 38)	38 (34, 43)	36 (33.5,39)	<0.001
Left atrial volume index (mL/m^2^)		35 (29, 40)	30.5 (29, 32)	39.5 (35, 47.3)	33 (29, 37.5)	53 (43, 55)	47.4 (37.5, 55)	<0.001
Left ventricle end-diastolic diameter index (mm/m^2^)		25 (22, 27)	28.5 (23, 34)	26 (23,28.6)	24 (22, 26.3)	22.5 (19, 25.2)	28.1 (25, 30.8)	<0.001
Mitral valve E/A ratio		0.7 (0.6, 0.8)	0.8 (0.6, 1)	1.1 (0.9, 1.2)	0.9 (0.7, 1.3)	3.7 (1.9, 4.8)	1.2 (0.8, 1.7)	<0.001
Average E/e’ ratio		12.8 (8.9, 15.6)	10.3 (7.5, 13)	15.1 (12, 17.5)	8.8 (7.8, 11.5)	21.6 (14.5, 27)	16.7 (14, 19.5)	<0.001
Elevated filling pressure (%)		21.7	0	80	26.5	100	91.3	<0.001
Diastolic dysfunction (%)	Moderate	8.7	0	71.7	24.5	57.1	72.5	<0.001
	Severe	0	0	4.3	0	42.9	11.6	
Meta-analysis Global Group in Chronic Heart Failure		23.5 (18.8, 25)	21.5 (21, 22)	24 (18, 28)	24 (15, 30)	13 (13, 13)	23 (18, 27.5)	0.780
Heart failure exacerbation (%)		17.4	0	28.3	8.1	85.7	46.4	<0.001
Cardiovascular mortality (%)		8.7	0	2.2	2	57.1	7.2	<0.001
All-cause mortality (%)		8.7	0	10.9	4.1	71.4	21.7	<0.001
Composite endpoints (%)		17.4	0	32.6	12.2	85.7	52.2	<0.001

*p*-value < 0.05 is statistically significant.

**Table 2:** Summary of clusters using the K-prototype method (*n* = 196).

Clusters		1	2	3	4	*p*-Value
Number of subjects		61	9	53	73	
Age (years)		80 (70.5, 87)	83 (72, 89.5)	84 (80, 87.5)	69 (61, 74.5)	<0.001
Male (%)		48	78	13	60	<0.001
Atrial fibrillation (%)		36	33	25	15	0.043
Hypertension (%)		67	78	77	75	0.603
Dyslipidemia (%)		56	33	38	59	0.065
Diabetes (%)		33	11	17	23	0.183
Coronary artery disease (%)		44	22	34	34	0.445
Chronic kidney disease (%)		25	33	25	8	0.029
Stroke or transient ischemic attack (%)		11	22	8	5	0.294
Lung disease (%)		7	11	9	8	0.934
Obstructive sleep apnea (%)		10	11	2	10	0.328
Body mass index (kg/m^2^)		26.6 (24.1, 31.5)	38.5 (28.5, 38.5)	23.1 (21.2, 25)	27.3 (24.1, 32.4)	<0.001
Systolic blood pressure (mmHg)		137 (121.5, 150)	115 (97, 134)	135 (123, 144)	132 (121, 144)	0.022
Low-density lipoprotein (mmol/L)		1.8 (1.2, 2.4)	2.1 (1.3, 2.4)	2.1 (1.6, 2.8)	2.2 (1.6, 2.9)	<0.034
Serum creatinine (mmol/L)		90 (78, 122.5)	94 (82, 147)	89 (68, 115)	88 (71, 98.5)	0.119
HbA1c (%)		5.9 (5.6, 6.4)	5.8 (5.4, 6.2)	5.8 (5.6, 6.2)	5.9 (5.6, 6.7)	0.651
B-type natriuretic peptide (pg/mL)		282 (111, 769)	817 (514, 1276)	128 (65, 515)	78 (25, 175)	<0.001
Meta-analysis Global Group in Chronic Heart Failure		23 (18, 27.8)	13 (13, 13)	24 (22, 29.5)	19 (12.3, 22)	<0.001
Left ventricular ejection fraction (%)		55 (53, 60)	57 (55, 60)	60 (60, 65)	60 (55, 60)	<0.001
Mild to Moderate mitral regurgitation (%)		76	89	85	53	<0.001
Mild to Moderate aortic stenosis (%)		14	11	22	8	0.089
Mild to Moderate aortic regurgitation (%)		22	44	57	11	<0.001
Mild to Moderate tricuspid regurgitation (%)		68	89	84	47	<0.001
Right ventricle diameter (mm)		36 (34, 40)	38 (34, 43)	34 (29, 37)	34 (30, 37)	<0.001
Left atrial volume index (mL/m^2^)		44 (36, 54)	53 (43, 56)	40 (35, 50)	33 (29, 39)	<0.001
Left ventricle end-diastolic diameter index (mm/m^2^)		27 (24, 29)	22.5 (19, 24.5)	28 (25, 30)	25 (22.5, 28)	<0.001
Mitral valve E/A ratio		1.2 (1, 1.9)	2.1 (1.7, 4.5)	0.8 (0.67, 1.1)	0.9 (0.7, 1.2)	<0.001
Average E/e’ ratio		15.8 (14.3, 19.6)	21.6 (15, 25.2)	16.7 (14, 19.3)	9.3 (7.9, 11.4)	<0.001
Pulmonary artery pressure (mmHg)		34 (27, 40)	48 (33, 49)	31 (24, 36)	26 (23, 29)	<0.001
Elevated filling pressure (%)		98	100	74	23	<0.001
Diastolic dysfunction	Moderate	80	67	60	19	<0.001
	Severe	15	33	0	1	
Heart failure exacerbation (%)		41	89	34	11	<0.001
Cardiovascular mortality (%)		5	67	4	3	<0.001
All-cause mortality (%)		21	78	9	5	<0.001
Composite endpoint (%)		48	89	38	14	<0.001

## Data Availability

No Applicable.
